# Grape Seed Flavanols Restore Peripheral Clock of White Adipose Tissue in Obese Rats Under Circadian Alterations

**DOI:** 10.3390/nu17223564

**Published:** 2025-11-14

**Authors:** María García-Martínez-Salvador, Marina Colom-Pellicer, Eliska Podolakova, Miquel Mulero, Gerard Aragonès, Jorge R. Soliz-Rueda, Begoña Muguerza

**Affiliations:** 1 Nutrigenomics Research Group, Biochemistry and Biotechnology Department, University Rovira i Virgili, 43007 Tarragona, Spain; maria.garciam@estudiants.urv.cat (M.G.-M.-S.); marina.colom@urv.cat (M.C.-P.); eliska.podolakova@estudiants.urv.cat (E.P.); miquel.mulero@urv.cat (M.M.); gerard.aragones@urv.cat (G.A.); begona.muguerza@urv.cat (B.M.); 2 Nutrigenomics Research Group, Institut d’Investigació Sanitària Pere Virgili (IISPV), 43204 Tarragona, Spain; 3 Nutrigenomics Research Group, Tecnología Ambiental Alimentaria y Toxicológica Center (TecnATox), 43007 Tarragona, Spain; 4 CIBERobn Physiopathology of Obesity and Nutrition, Institute of Health Carlos III, 28029 Madrid, Spain

**Keywords:** chrononutrition, circadian rhythms, metabolism, phenolic compounds, photoperiod, *zeitgeber*

## Abstract

**Background**: White adipose tissue (WAT) exhibits diurnal oscillations regulated by clock genes, which autonomously control its functionality. These rhythms are modulated by the central clock and external factors, such as light exposure and diet. Flavanols, phenolic compounds known for their beneficial metabolic effects, have been shown to modulate the expression of clock genes. This study explored the impact of flavanols on clock gene expression in WAT explants from lean and obese rats under changes in light/dark cycles. **Methods**: WAT explants were obtained from 24 Fischer rats fed a standard diet (STD) or cafeteria diet (CAF) for seven weeks. During the final week, rats were changed to short (6 h of light, L6) or long (18 h of light, L18) photoperiods. CAF-fed rats were also administered a grape seed (poly)phenol-rich extract (GSPE) (25 mg/kg) or vehicle (VH). After sacrifice, WAT explants were collected every 6 h starting at 8 a.m. the following day (CT0, CT6, CT12, CT18, and CT24). **Results**: The results showed that under L18 conditions, STD-fed rats displayed oscillations in *Bmal1*, *Cry1*, *Per1*, and *Rev-erbα* clock gene expression, whereas many of these rhythms were disrupted under L6 conditions. Moreover, the administration of the CAF diet also resulted in the loss of clock gene circadian oscillations in the WAT explants. GSPE administration restored the oscillation of these clock genes under L18 and L6 conditions. **Conclusions**: These findings highlight the potential *zeitgeber* role of flavanols in modulating WAT peripheral clocks and their capacity to improve metabolic and circadian regulation under conditions of diet- and photoperiod-induced disruption.

## 1. Introduction

Circadian rhythms are endogenous oscillations that last approximately 24 h, regulating essential physiological processes, including sleep–wake cycles, metabolism, gastrointestinal functions, endocrine functions, and behavior [1,2]. Organisms are composed of different internal clocks around the tissues, and all of them are controlled by the central clock that acts as a pacemaker [3]. This master clock is located in the hypothalamic suprachiasmatic nucleus (SCN) and its activity is regulated by the dimerization of ARNT-like 1 (BMAL1) and circadian locomotor output cycles kaput (CLOCK), proteins which activate the transcription of period family members (*Per1*, *Per2*, *Per3*), cryptochrome family members (*Cry1, Cry2*) and the nuclear receptor subfamily 1 group D members 1 and 2 (*Rev-erbα*, *Rev-erbβ*) [1,3]. In turn, PER and CRY proteins form a dimer inhibiting the transcription of *Bmal1* and *Clock*, creating a negative feedback loop. Additionally, REV-ERBα also inhibits the transcription of *Bmal1*. When the levels of BMAL1 and CLOCK proteins decrease, *Per*, *Cry*, and *Rev-erbα* slow down the transcription rate, avoiding the repression of *Bmal1* and *Clock* expression and activating them again [1,3]. Other gene expressions are enhanced by the CLOCK/BMAL1 heterodimer, such as nicotinamide phosphoribosyltransferase (*Nampt*). This gene plays a pivotal role in regulating sirtuin (SIRT1) activity which exerts an influence on the biological clock, acting as a link between the energy metabolism and clock genes [4,5]. This circadian system is characterized by its autonomy, since it can work without any external stimuli [1]. However, external cues, also called *zeitgebers*, can help to synchronize this system to the environment [3,6]. In fact, organisms can respond to different environmental stimuli such as temperature, eating, exercise, social interactions, and light, with this last *zeitgeber* being the most important [1]. The light/dark cycle works as a functional biological clock manager and controls the feedback cycles at the transcriptional and post-translational levels through the activation and repression of participating genes [1,6]. Nevertheless, light not only refers to the time of the day but also concerns the changes caused by the Earth’s rotation around the Sun, generating seasonal changes and promoting metabolic adaptations [3]. Mammals respond differently to photoperiods, either long or short, changing their body weight (BW) where white adipose tissue (WAT) metabolism is involved [7]. In fact, it has been reported that a change in photoperiod, especially to a short photoperiod, causes circadian alterations and can alter lipid metabolism, promoting obesity in healthy rats [8].

Current lifestyles, characterized by overexposure to artificial light, shift works, social jetlag and excessive food intake, exacerbate circadian misalignment, contributing to the development of metabolic syndrome (MetS) and the emerging concept of “circadian syndrome” [9,10,11,12,13]. Circadian syndrome includes a cluster of metabolic and physiological disorders, including obesity, dyslipidemia, insulin resistance, cognitive dysfunction, and sleep disturbances, all driven by disruptions in circadian gene regulation. Obesogenic diets, such as the cafeteria (CAF) diet, typified by ultra-processed, energy-dense foods, have been shown to impair energy metabolism, promote inflammation, and exacerbate oxidative stress [14,15]. These dietary patterns also disrupt diurnal oscillations of clock genes and metabolic pathways, reinforcing the association between circadian misalignment and MetS [12,16,17,18,19]. In addition, different studies suggest that the combined effects of obesogenic diets and altered photoperiods synergistically worsen metabolic and behavioral outcomes in animal models [20]. In fact, the negative effect of a sudden photoperiod change is further strengthened if it is accompanied by a CAF diet [21,22,23]. These effects are visible from BW differences to differences in gene expression. However, the impact of alterations in light/dark cycles on specific tissues, such as subcutaneous inguinal WAT (iWAT), remains poorly understood. As a metabolically active depot, iWAT plays a critical role in energy homeostasis and demonstrates sensitivity to environmental and circadian cues [24,25]. Despite its relevance, no study has explored circadian gene oscillations in iWAT explants under changes in the light/dark cycles and in an obesogenic context, a methodology that allows tracking of gene expression dynamics across the circadian cycle.

Interestingly, it has been recently observed that bioactive compounds, such as flavanols, can play a *zeitgeber* role by influencing clock gene expression [26], acting as agonists of nuclear receptors [27] or via post-translational [28] and epigenetic [29] modifications. Flavanols, a sub-class of flavonoids that are abundant in several foods, such as wine, tea, cocoa, and grape seeds, have also been closely related to the mitigation of chronic non-communicable diseases such as obesity or MetS, both also related to circadian rhythm function as mentioned above [26]. In addition, the consumption of these compounds can contribute to the adaptation of organisms to environmental conditions, which have also been seen to influence adipose tissue [30]. Interestingly, our group has recently demonstrated how flavanols could mitigate the effects produced by a light/dark cycle change in short or long photoperiods in both healthy and obese rats [21,22,23], showing differences in BW gain, lipid metabolism, and energy intake [21]. Indeed, serum biochemical parameters, serum metabolites, and hepatic antioxidant response were improved with flavanol administration after an abrupt photoperiod change but their effects differed depending on the photoperiod conditions, being more visible in long photoperiods [22,23]. These differences could be promoted by the clock gene modulation in both central and peripheral clocks [26]. In this regard, our group reported that grape seed flavanols upregulated the *Per1* gene, while the *Rev-erbα* and *Rorα* genes were downregulated in mesenteric WAT from healthy and obese rats [26]. In addition, we have recently demonstrated in iWAT explants from obese PERIOD2::LUCIFERASE (PER2::LUC) circadian reporter mice that serum metabolites derived from flavanols increased the amplitude and period of the circadian oscillations of the *Per2* gene compared to non-treated explants [31]. Interestingly, the modulation of lipogenesis and lipolysis gene expression by flavanols was also observed [31].

Therefore, the aim of this study was to evaluate the state of the iWAT peripheral clock after a light/dark cycle change in healthy and obese rats and to study whether flavanols can restore the deleterious effects of a CAF diet on WAT under light/dark cycle changes, and their potential role in recovering circadian rhythms. For this purpose, iWAT tissue explants were obtained from 24 Fischer 344 Rats which were exposed to a 12 h light/dark cycle and fed a standard (STD) diet or CAF diet for 7 weeks. In the seventh week, animals were changed to short (6 h of light and 18 h of darkness, L6) or long (18 h of light and 6 h of darkness, L18) photoperiods and administered grape seed (poly)phenol-rich extract (GSPE) (25 mg/kg animal) or vehicle (VH). After this week, animals were sacrificed and explants from iWAT were maintained in cell culture medium. Then, samples were collected every 6 h until a 24 h cycle was completed to analyze tissue circadian oscillation.

## 2. Materials and Methods

### 2.1. Grape Seed (Poly)Phenol-Rich Extract

The extract of flavanols used in this study was obtained from white grape seeds and provided by Les Dérives Résiniques et Terpéniques (Dax, France). The phenolic profile was composed of catechin, epicatechin, gallic acid, and epicatechin gallate, and dimers, trimers, and tetramers of proanthocyanidins. The phenolic composition of this extract was previously analyzed by our group [32].

### 2.2. Animal Experiment Procedure

Twenty-four 13-week-old male F344/IcoCrl rats from Charles Rives Laboratories (Barcelona, Spain) were housed individually for 2 weeks under STD conditions of 22 °C, and 12 h light/dark cycle (L12), 350 lux of light density, and ad libitum access to food and water. Lights were switched on at 8 a.m. and switched off at 8 p.m. After this period of adaptation, animals were weighted and randomly grouped into two groups. One group were fed a STD chow diet composed of 72% carbohydrate, 8% lipid, and 19% protein (diet administered by Safe-A04c, Scientific Animal Food and Engineering, Barcelona, Spain), while the other group were fed with a CAF diet for 7 weeks. This diet contained bacon, cookie with *paté*, cookie with cheese, carrot, *ensaïmada* (pastry), STD chow, and sweetened milk (22% sucrose (*w*/*v*)) and overall, it was composed of 55% carbohydrate, 33.6% lipid, and 11.4% protein.

In the last week, animals were transferred into two adjacent similar rooms, which differed only in the light/dark cycle duration, L18 photoperiod or L6 photoperiod, and administered GSPE (*n* = 4 per CAF-GSPE groups in each photoperiod) or VH (*n* = 4 per STD and CAF-VH groups in each photoperiod) as described previously (Figure S1) [21,22,23]. The dose of flavanol extract was 25 mg/kg animal, which is estimated to be a dose of 370 mg/day for a 70 kg human [33]. The light intensity was 350 lux for both photoperiods. For the L6 photoperiod, the lights were turned on at 8 a.m. and turned off at 2 p.m., whereas for the L18 photoperiod, the lights were turned on at 8 a.m. and turned off at 2 a.m. the following day. To minimize the effect of human intervention and to not interrupt the light/dark conditions, all daily and non-invasive procedures were conducted in the same room to ensure equal treatment for all animals, while more complex procedures were performed in a separate room for a short period of time and under required light conditions. *Zeitgeber* time (ZT) 0 was determined at 8 a.m. when the dose was administered. The general health, BW changes, grooming, and behavior of the animals were monitored daily. No procedures were performed that could cause pain or distress beyond those resulting from routine handling and oral administration. Therefore, no specific analgesic regimen was necessary. Any animal showing signs of pain, distress, or rapid weight loss would have been examined by the designated veterinarian and withdrawn from the study, if necessary, as stipulated in the approved protocol.

Animal procedures were approved by The Animal Ethics Committee of the University Rovira i Virgili (Tarragona, Spain) and the Generalitat de Catalunya (reference number 9495, 18 September 2019) and were carried out in accordance with Directive 86/609EEC of the Council of the European Union.

### 2.3. Sample Collection

After the experimental period, animals were food-deprived for 3 h and then anesthetized with 3% isoflurane prior to sacrifice by decapitation. For the analysis of this study, iWAT explants were collected from CAF-fed animals administered with VH or GSPE under the two photoperiods (*n* = 4/group). Samples from the STD-VH groups (*n* = 4/group) were collected as a control for each photoperiod [21,22,23]. Sacrifices were conducted between ZT4 and ZT5, approximately 4 h after the last dose was administered, to ensure consistency in gene oscillatory states across all samples. Immediately after sacrifice, the iWATs were removed and weighed. Samples of 300 mg of iWAT from each animal were split and cultured in 6-well plates under controlled conditions (7% CO_2_) in Dulbecco’s Modified Eagle Medium (DMEM), supplemented with 10% fetal bovine serum (Thermo Fisher Scientific Life Sciences, Waltham, MA, USA), glucose (4.5 g/L), and a mixture of penicillin–streptomycin–glutamine (Thermo Fisher Scientific Life Sciences). Sample collection started the next day at 8 a.m., corresponding to Circadian Time 0 (CT0). Samples were collected every 6 h during a 24 h cycle, resulting in five time points from each animal sample (*n* = 4 per group), CT0, CT6, CT12, CT18, and CT24, to analyze the circadian oscillation of clock genes under changes in light/dark cycles. Collected samples were immediately stored at −80 °C for subsequent analysis.

### 2.4. Gene Expression Quantification

For the RNA isolation, samples were mixed with TRIzol™ Reagent (Thermo Fisher, Madrid, Spain). After homogenization with Tissue Lyser LT (Qiagen, Madrid, Spain), the homogenate was centrifuged for 10 min at 12,000× *g* at 4 °C and the resulting supernatant was transferred to a new tube. Then, 250 µL of chloroform was added and centrifuged for 15 min at 12,000× *g* and 4 °C, obtaining an aqueous phase that was transferred to a new tube. A total of 500 µL of isopropanol was added and centrifuged for 10 min at 12,000× *g* and 4 °C to obtain the mRNA pellet, which was washed twice with 70% ethanol and 5 min centrifugations at 8000× *g* and 4 °C. Once the pellet was washed and dried, it was resuspended in 20 µL of nuclease-free water. The RNA yield was quantified on a NanoDrop 1000 spectrophotometer (Thermo Scientific, Wilmington, DE, USA).

The High-Capacity cDNA Reverse Transcription kit (Thermo Fisher Scientific, Wilmington, DE, USA) and Labnet MultiGene Gradient PCR Thermal Cycler (Sigma_Aldrich, Madrid, Spain) were used to synthesize cDNA. Relative gene expression was measured with NZYSupreme qPCR Green Master Mix (NZYTech, Lisbon, Portugal) and a QuantStudioTM 5 Real-Time PCR Instrument (Thermo Fisher Scientific, Wilmington, DE, USA). The fold changes in the mRNA levels were calculated by normalizing to L6-STD in the CT0 group using the 2^−∆∆Ct^ method with the Peptidylprolyl isomerase A (*Ppia*) gene as an endogenous control, as reported by Schmittgen and Livak [34]. This gene has not shown variation in the CTs obtained across the five time points, demonstrating good consistency as a housekeeping gene for analyzing the oscillation of target genes. The primers used for each gene are shown in Table S1 and were obtained from Biomer.net (Ulm, Germany).

### 2.5. Statistical Analysis

In order to analyze oscillation of gene expression, and taking into account the dependent data of our experiment, a mixed-effects cosinor model (cosinor method) that includes random intercepts to capture intra-subject correlation was used [35]. This method makes it possible to calculate the diurnal oscillation rhythmicity parameters over a 24 h period, such as mesor (mean adjusted to the diurnal rhythm), amplitude (difference between the maximum value and the mean value of a wave), or acrophase (time at which the peak of a rhythm occurs). A 24 h period is assumed given the characteristics of the study. Although there may be small deviations around 24 h in the oscillation of gene expression, these could not be considered due to the low frequency of time points available (every 6 h in 24 h), which would not allow these variations to be studied. The presence of circadian oscillations was considered when the model of the expressions of each gene fitted the cosine curves (amplitude ≠ 0 and *p amplitude* < 0.05). For this analysis, the *GLMMcosinor* package was used (https://github.com/ropensci/GLMMcosinor (accessed on 25 October 2025)). Our main hypothesis is whether genes show rhythmicity in explants from obese animals. The remaining comparisons are secondary or exploratory hypotheses. Due to the limited number of comparisons, the *p*-values presented in this study are without FDR adjustment. The Kruskal–Wallis test or Mann–Whitney test were used to analyze the data, as indicated in the respective figure legends. These statistical analyses were performed using the statistical software package SPSS Statistics 22 (SPSS Inc., Chicago, IL, USA).

Sample size was estimated with a multi-regression power analysis in G Power software (Düsseldorf, Germany) using prior data on melatonin levels [21] as the reference effect, an alpha of 0.05, and a target power of 80%. To ensure better accuracy in estimating the oscillatory parameters, we sampled five equidistant time points across a 24 h period. This satisfies the Nyquist theorem [36]. CT0 and CT24 represent the same time point, repeated at the beginning and end of the measurement, respectively. Previous studies have also shown that both the sample size and the number of time points are adequate for analyzing oscillations over a 24 h period [17,37,38].

## 3. Results

### 3.1. Effects of Grape Seed Flavanols on Photoperiod-Dependent Changes in Body Weight

**Figure 1:**
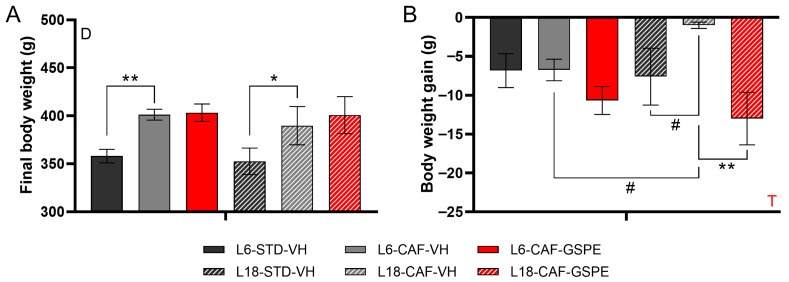
(**A**) Final body weight and (**B**) body weight gain during the last week of the experiment for L6 and L18 conditions (STD-VH, CAF-VH and CAF-GSPE) in the seventh week. D, diet effect; T, GSPE administration effect. * or ** indicate significant differences between groups (*p* ≤ 0.05 and *p* ≤ 0.01, respectively) and # indicates tendency (*p* = 0.1–0.051) using 2-way ANOVA followed by LSD post hoc test. STD, standard diet-fed rats; CAF, cafeteria diet-fed rats; VH, rats administered vehicle; GSPE, rats administered 25 mg/kg grape seed (poly)phenol extract; L6, short photoperiod, 6 h light per day; L18, long photoperiod, 18 h light per day.

As expected, rats fed a CAF diet showed significantly higher BW than those fed an STD diet under both photoperiod conditions at the end of the experimental period (*p* = 0.008 and *p* = 0.031, L6 and L18, respectively) (Figure 1A). The change in light/dark cycle caused a loss of BW gain in all experimental groups (Figure 1B). A tendency to lose less BW was observed for animals fed the CAF diet compared to animals fed the STD diet (*p* = 0.088) when they were transferred to the L18 photoperiod. In addition, the administration of GSPE after a switch to the L18 photoperiod resulted in a substantial loss of BW gain compared to the CAF-VH group (*p* = 0.001). Interestingly, a photoperiod effect was observed in the CAF-VH groups, as this group tended to lose greater BW gain under L6 conditions compared to the L18-CAF-VH group (*p* = 0.021). No changes were observed after GSPE administration in BW gain under L6 conditions (Figure 1B).

### 3.2. Change to L6 Photoperiod Affects Circadian Expression of Core Clock Genes in White Adipose Tissue

**Figure 2:**
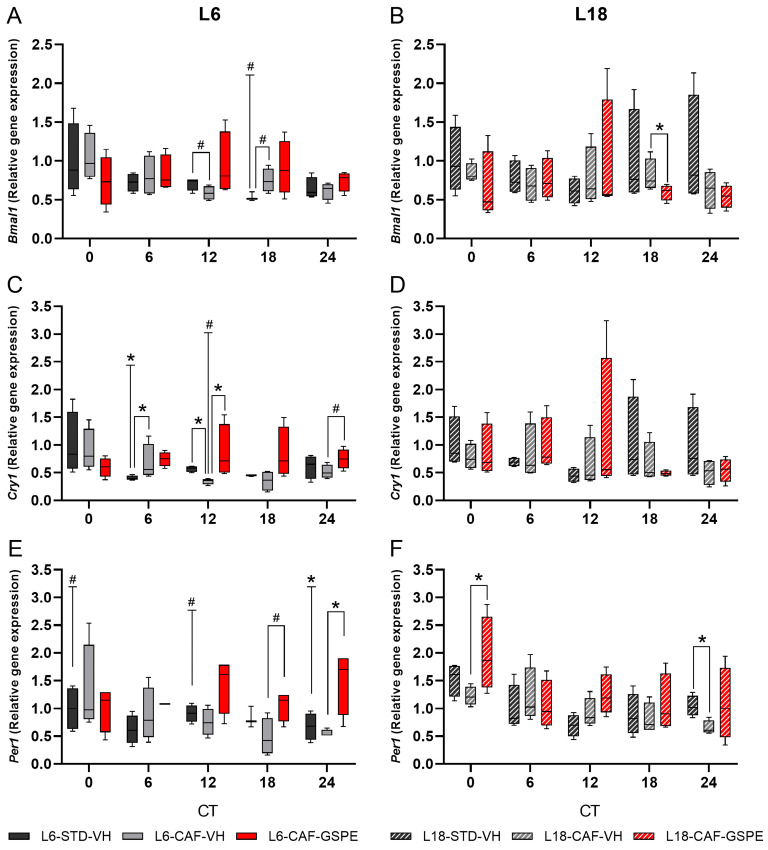
Relative gene expression of core clock genes in inguinal white adipose tissue (iWAT). *Bmal1*, *Cry1*, and *Per1* relative gene expressions in iWAT under L6 (**A**,**C**,**E**) and L18 (**B**,**D**,**F**) conditions. * indicates significant differences (*p* < 0.05) and # tendency (*p* = 0.1–0.051) between groups at each time point by Mann–Whitney test. Significant differences or trends between groups by photoperiod are indicated in the L6 graphs above the T symbol. STD, standard diet-fed rats; CAF, cafeteria diet-fed rats; VH, rats administered vehicle; GSPE, rats administered 25 mg/kg grape seed (poly)phenol extract; L6, short photoperiod, 6 h light per day; L18, long photoperiod, 18 h light per day; CT, circadian time.

**Figure 3:**
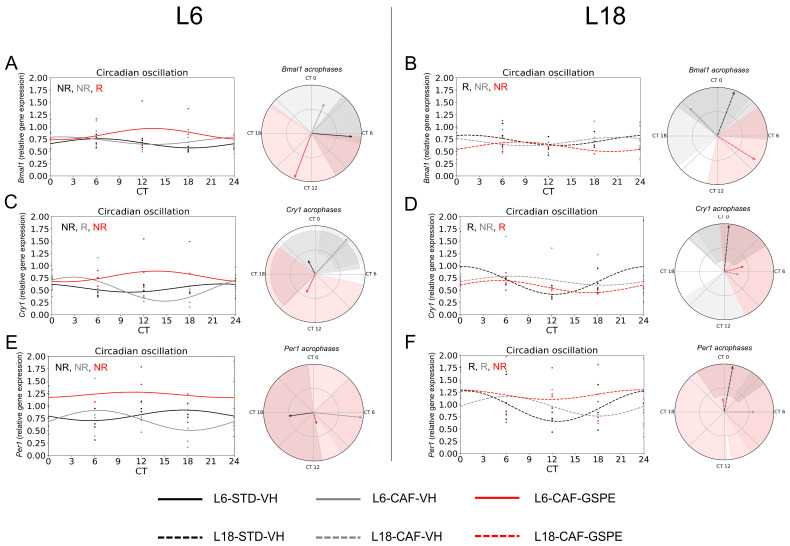
Estimated circadian oscillations of core clock genes in inguinal white adipose tissue. Estimated circadian rhythms and acrophases (indicated by the arrows) with their amplitude (indicated by the arrow size) represent *Bmal1* genes for STD-VH, CAF-VH, and CAF-GSPE under (**A**) L6 and (**B**) L18 conditions. Estimated circadian rhythms and acrophases with their amplitude represent *Cry1* genes for STD-VH, CAF-VH, and CAF-GSPE under (**C**) L6 and (**D**) L18 conditions. Estimated circadian rhythms and acrophases with their amplitude represent *Per1* genes for STD-VH, CAF-VH, and CAF-GSPE under (**E**) L6 and (**F**) L18 conditions. STD, standard diet-fed rats; CAF, cafeteria diet-fed rats; VH, rats administered vehicle; GSPE, rats administered 25 mg/kg grape seed (poly)phenol extract; L6, short photoperiod, 6 h light per day; L18, long photoperiod, 18 h light per day; CT, circadian time; R, significant or tends to rhythmic; NR, non-rhythmic.

To evaluate the effects of photoperiod, diet, and GSPE administration on the circadian oscillation of peripheral clock genes in iWAT explants, relative gene expression was quantified for core clock genes. The cosinor method was used to model the oscillatory behavior of these genes over a 24 h period. Circadian parameters, including amplitude, acrophase, and mesor, were obtained for individual groups. For *Bmal1* gene expression, photoperiod effects showed a trend for decreased expression under L6 conditions compared to L18 conditions at CT18 in STD-fed animals by Mann–Whitney test (*p* = 0.077) (Figure 2A). Under L6 conditions, diet effects revealed trends toward reduced expression in CAF diet-fed animals at CT12 (*p* = 0.083) and increased expression at CT18 (*p* = 0.077) compared to STD-fed rats by Mann–Whitney test, whereas no significant differences were detected under L18 conditions (Figure 2B). Regarding GSPE administration effects, significant differences were observed under L18 conditions, reducing expression with GSPE administration at CT18 (*p* = 0.043) (Figure 2B) by Mann–Whitney test. Regarding the rhythmicity analysis by the cosinor method, under STD-fed conditions, only L18-STD group showed a tendency for rhythmicity (*p* = 0.083), while in CAF-fed conditions, any group presented rhythmic oscillations. In relation to GSPE administration, L6-CAF-GSPE exhibited a tendency to display rhythmicity (*p* = 0.060) (Table S2, Figure 3A,B).

Regarding *Cry* gene expression, a decrease in gene expression caused by the photoperiod effect was observed for the STD group under L6 conditions compared to the L18-STD-VH group at CT6 (*p* = 0.021), and a tendency to reduce expression in CAF-fed rats at CT12 by Mann–Whitney test (*p* = 0.083) (Figure 2C). Regarding diet effects, significant differences were found in the L6 conditions, with increased expression observed in CAF-fed rats at CT6 (*p* = 0.043) and increased expression at CT12 by Mann–Whitney test (*p* = 0.021) (Figure 2C). For GSPE administration effects, an increase in gene expression under L6 conditions was detected at CT12 (*p* = 0.034), and a tendency to increase at CT24 by Mann–Whitney test (*p* = 0.077) (Figure 2C). No differences were found under L18 conditions by Mann–Whitney test (Figure 2D). Rhythmicity analysis revealed that the L6-STD group showed no rhythmicity (Table S3, Figure 3C), while the L18-STD group showed significant oscillation when the cosinor method was used (*p* = 0.001) (Table S3, Figure 3D). Regarding the CAF groups, although rhythmicity was observed in the L6-CAF-VH group (*p* = 0.007), no rhythmicity was found in the L18-CAF-VH group (Table S3). Additionally, no rhythmicity was found in the group under L6 conditions after GSPE administration. In contrast, under L18 conditions, the GSPE-administered group showed a tendency to restore rhythmicity by cosinor method (*p* = 0.088) (Table S3), which was missing in the L18-CAF-VH group.

For *Per* gene expression, photoperiod effects revealed a decrease in gene expression under L6 conditions compared to the L18 group between the STD-fed groups at CT24 (*p* = 0.043) (Figure 2E). In addition, a trend toward reduced expression at CT0 (*p* = 0.083) and increased expression at CT12 was observed by the Mann–Whitney test (*p* = 0.083) (Figure 2E). Regarding diet effects, reduced gene expression was detected in CAF-fed groups compared to STD-fed animals under L18 conditions at CT24 (*p* = 0.043, Figure 2F). GSPE administration under L6 conditions resulted in an increase in gene expression at CT24 (*p* = 0.034) and a tendency to increase gene expression at CT18 (*p* = 0.077) (Figure 2E), whereas an increase in gene expression at CT0 was observed under L18 conditions by the Mann–Whitney test (*p* = 0.043, Figure 2F). The analysis of rhythmicity revealed that only the STD-fed and CAF-fed group under L18 conditions showed rhythmicity (*p* = 0.001) and a trend to be rhythmic (*p* = 0.082), respectively (Table S4, Figure 3E,F). In contrast, none of the other groups showed oscillation (Figure 3E,F, Table S4).

### 3.3. Rev-Erbα and Nampt Exhibited No Rhythmicity in CAF-Fed Groups, While Grape Seed Flavanols Restored Rev-Erbα Oscillation Disrupted by the CAF Diet Under L18 Conditions

**Figure 4:**
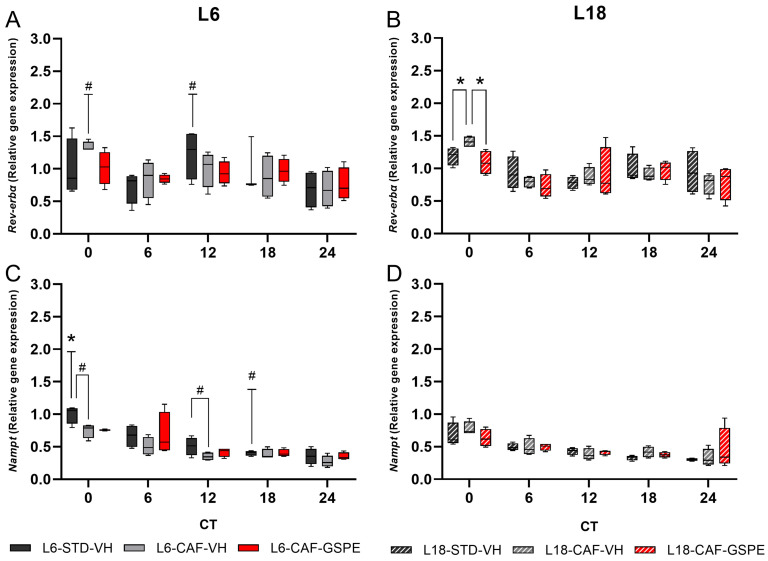
Relative gene expression of clock-controlled genes in inguinal white adipose tissue. *Rev-erbα* relative gene expression in iWAT under (**A**) L6 and (**B**) L18 conditions. *Nampt* relative gene expression in iWAT under (**C**) L6 and (**D**) L18 conditions. * indicates significant differences (*p* < 0.05) and # indicates the tendency (*p* = 0.1–0.051) between groups at each time point by Mann–Whitney test. Significant differences or trends between groups by photoperiod are indicated in the L6 graphs above the T symbol. STD, standard diet-fed rats; CAF, cafeteria diet-fed rats; VH, rats administered vehicle; GSPE, rats administered 25 mg/kg grape seed (poly)phenol extract; L6, short photoperiod, 6 h light per day; L18, long photoperiod, 18 h light per day; CT, circadian time.

**Figure 5:**
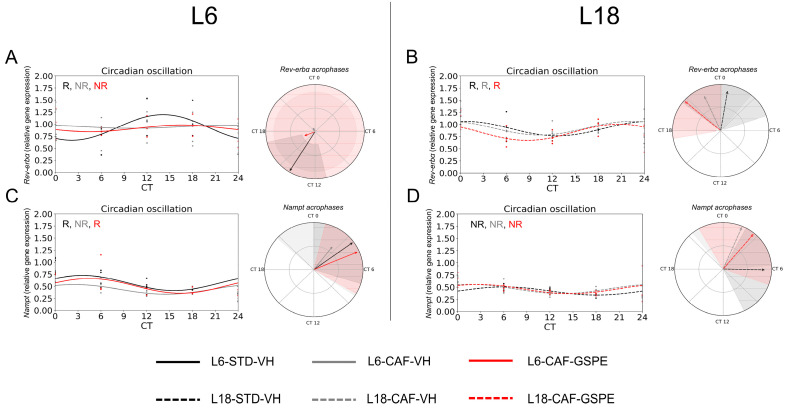
Estimated circadian oscillations of clock-controlled genes in inguinal white adipose tissue. Estimated circadian rhythms and acrophases (indicated by the arrows) with their amplitude (indicated by the arrow size) representing *Rev-erbα* genes for STD-VH, CAF-VH, and CAF-GSPE under (**A**) L6 and (**B**) L18 conditions. Estimated circadian rhythms and acrophases with their amplitude representing *Nampt* genes for STD-VH, CAF-VH, and CAF-GSPE under (**C**) L6 and (**D**) L18 conditions. STD, standard diet-fed rats; CAF, cafeteria diet-fed rats; VH, rats administered vehicle; GSPE, rats administered 25 mg/kg grape seed (poly)phenol extract; L6, short photoperiod, 6 h light per day; L18, long photoperiod, 18 h light per day; CT, circadian time; R, significant or tends to rhythmic; NR, non-rhythmic.

In order to investigate the influence of the changes in the core clock genes on the clock-controlled genes (CCGs) and the implications it could have on the energy metabolism, we analyzed *Rev-erbα* and *Nampt* genes. For *Rev-erbα*, relative gene expression tended to decrease in CAF-fed rats under L6 conditions compared to L18 at CT0 (*p* = 0.083) and a tendency to increase expression at CT0 was seen with the Mann–Whitney test (*p* = 0.083) (Figure 4A). Regarding diet effects, increased gene expression was observed in rats fed the CAF compared to STD under L18 conditions at CT0 by the Mann–Whitney test (*p* = 0.021), while GSPE administration resulted in a decrease in gene expression at this time point, returning it to STD values (*p* = 0.021) (Figure 4B). Regarding the circadian oscillation of *Rev-erbα*, the STD-fed groups under both photoperiods showed significant oscillation (*p* = 0.001 for L6-STD-VHand *p* = 0.039 for L18-STD-VH) (Table S5, Figure 5B) by cosinor method. Moreover, the acrophases of these oscillations are completely opposite (Figure 5A,B). In contrast, under CAF-fed conditions, only the L18-CAF-VH group presented a trend of rhythmicity (*p* = 0.057) (Figure 5A,B).

Under L6 conditions, GSPE administration did not restore the lost rhythmicity in CAF-fed rats (Table S5, Figure 5A). Interestingly, under L18 conditions, the L18-CAF-GSPE group showed a significant rhythmic oscillation (*p* = 0.037) (Table S5, Figure 5B). Regarding *Nampt* gene expression, photoperiod effects in STD-fed animals resulted in increased gene expression under L6 conditions compared to L18 in CT0 (*p* = 0.043) and a tendency for increased expression in CT18 by the Mann–Whitney test (*p* = 0.077) (Figure 4C,D). In addition, a trend towards lower expression was observed in animals fed CAF under L6 conditions at CT0 (*p* = 0.083) and CT12 by the Mann–Whitney test (*p* = 0.083) (Figure 4C). However, no significant differences after GSPE administration were observed in any of the photoperiodic conditions by the Mann–Whitney test (Figure 4C,D). The rhythmicity analysis by the cosinor method for *Nampt* revealed that the L6-STD group (Table S6, Figure 5C) showed rhythmicity (*p* = 0.024), while the L18-STD group (Table S6, Figure 5D) showed no rhythmicity. For the CAF-fed and GSPE-administered groups, L6-CAF-GSPE restored the rhythmicity (*p* = 0.047) lost in the L6-CAF-VH group, while rhythmicity was detected in all L18-CAF groups by the cosinor method (Table S6, Figure 5C,D).

## 4. Discussion

Modern sedentary lifestyles, overexposure to artificial light, and unhealthy dietary habits, among other factors, are strongly associated with MetS [1,9]. Indeed, a key factor contributing to MetS is biological rhythm disruption, often accompanied by impaired peripheral clocks such as WAT metabolism [9,24,25,39,40,41]. In this regard, light plays a crucial role in the synchronization of core clock genes, which regulate metabolic and physiological functions to adapt to environmental changes. In fact, different studies have shown that variations in day length can influence BW, metabolism, and core clock gene expression, highlighting the critical role of light in circadian regulation [8,42,43]. Interestingly, flavanols have demonstrated beneficial properties to mitigate these circadian and metabolic disturbances, offering a potential therapeutic approach [16,17,21,22,26,30]. In this study, we are the first to analyze the state of the peripheral clock in the WAT of animals fed STD or CAF diets and exposed to light/dark cycle changes by analyzing the oscillation of gene expression in iWAT explants. Furthermore, we evaluated the potential role of grape seed flavanols as a *zeitgeber* in CAF-fed animals. The methodology used in this study was based on previous research analyzing the oscillatory behavior of subcutaneous WAT clock genes in humans [44]. Although tissue explants lose their direct connection to the SCN, it is well established that explants can retain intrinsic rhythmicity for 48–72 h under controlled conditions. This behavior has been observed not only in human adipose tissue [45], but also in other tissues, such as the retina [46] and rat adrenal glands [47], highlighting the viability of using tissue explants to study peripheral circadian rhythms.

In this study, it was described that all groups tended to lose BW when they were changed to a long or short photoperiod. Only rats fed the CAF diet showed significant changes in BW gain due to photoperiod and photoperiod-dependent GSPE effects [21]. In addition, the results showed that under STD diet conditions, rats that were switched to an L6 photoperiod exhibited a loss of rhythmicity in almost all clock genes in iWAT, whereas when switching to an L18 photoperiod, most genes maintained or tended to maintain their rhythmicity except *Nampt*. It is well established that young rats bred under a L12 photoperiod perceive this light/dark cycle as a long photoperiod. Previous studies have shown that photoperiods with 14 or 16 h of light produce similar effects to L12 photoperiods, whereas significant differences were observed under shorter photoperiods with 10 or 8 h of light [48]. Accordingly, the change from L12 to L18 did not represent a significant change in circadian oscillation in the major clock genes, whereas the change to an L6 photoperiod markedly impacted the rhythmicity of the iWAT clock genes. In contrast, rats fed a CAF diet exhibited a complete loss of rhythmicity in the iWAT core clock genes under the L6 and L18 photoperiods. The CAF diet is widely used in animal studies because of its ability to induce metabolic disorders that mimic the effects of a human obesogenic diet, including obesity, dyslipidemia, and insulin resistance [14,15]. In addition to its metabolic effects, the CAF diet has been shown to alter circadian gene expression in several peripheral tissues, including liver clock genes in rats [26], maternal adipose clock genes during pregnancy [49], and hippocampal nucleus clock genes in a mouse model [50]. Additionally, diurnal metabolic disruption of iWAT has been observed in F344 rats subjected to a CAF diet [16]. In agreement with these results, our findings revealed that the CAF diet altered the rhythmicity of key iWAT clock genes, including *Per1*, *Cry1*, *Rev-erbα*, and *Bmal1*, compared with STD-fed rats under L18 photoperiod conditions.

**Figure 6:**
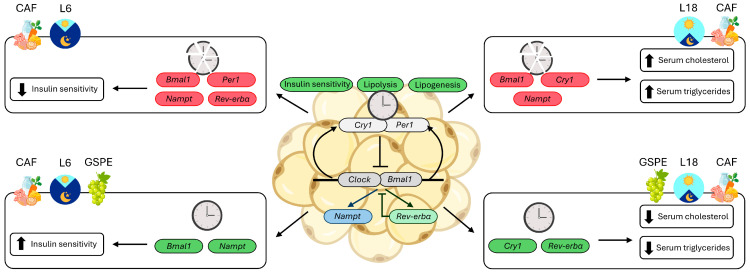
Summary diagram of the relationship between clock genes and observed effects. The proposed pathways that are altered by the CAF diet and that could promote detrimental effects on the peripheral clock of iWAT and metabolism, depending on the photoperiod to which the animals were exposed. In addition, the possible clock genes involved in the beneficial effects of GSPE on the damage caused by the CAF diet are shown. Under L6 conditions, lower insulin sensitivity was observed in obese rats and iWAT explants showed a loss of circadian oscillation in *Bmal1*, *Per1*, *Nampt*, and *Rev-erbα*. Obese rats under L18 conditions displayed a worsened serum lipid profile, while iWAT explants from these animals showed circadian disruption in genes such as *Bmal1*, *Cry1*, and *Nampt*. With GSPE supplementation, the animals showed improved insulin sensitivity under L6 conditions, exhibiting circadian rhythmicity in genes such as *Bmal1* and *Nampt*. These genes are closely related to insulin sensitivity [52]. GSPE supplementation under L18 conditions improved cholesterol and triglyceride levels, while their explants showed rhythmicity in clock genes, such as *Cry1* and *Rev-erbα*, involved in the regulation of lipid metabolism [53,54,55,56], which could suggest better circadian regulation of pathways such as lipolysis and lipogenesis. CAF, cafeteria diet-fed rats; GSPE, rats administered 25 mg/kg grape seed (poly)phenol extract; L6, short photoperiod, 6 h light per day; L18, long photoperiod, 18 h light per day.

GSPE has been widely described to mitigate metabolic disturbances caused by obesogenic diets or changes in photoperiod [30]. Our group have demonstrated protective effects of GSPE on different tissues and comorbidities, including its interaction with the circadian clock. Specifically, in iWAT, GSPE administration reduced the size of adipocytes and increased their number in CAF-fed rats [51]. Furthermore, GSPE was able to improve the circadian metabolomic profile in iWAT in CAF diet-fed rats [51]. We have recently demonstrated that GSPE supplementation at the trough was able to increase the rhythmicity of *Per2* expression in iWAT explants from obese PER2::LUC circadian reporter mice, while increasing the expression of lipid metabolism genes. This suggests that GSPE’s ability to improve the lipid profile may be linked to its ability to interact with core clock genes in iWAT [31]. In the present experimental model, GSPE mitigated the stress associated with light/dark cycle changes by attenuating corticosterone increases under L18-STD and L6-CAF conditions. It also reduced BW gain in CAF-fed rats compared with the groups receiving VH and improved testosterone concentrations under L18 conditions [21]. Regarding serum parameters, GSPE administration decreased cholesterol levels, aligning them with those of STD-fed rats, and reduced triglyceride levels in CAF-fed rats under L18 conditions (Figure 6). In addition, GSPE mitigated variations in serum metabolites caused by CAF diets. Finally, GSPE has been shown to increase hypothalamic *Nampt* expression in L18 conditions compared to CAF-fed rats [22].

In iWAT explants, the *Cry1* and *Rev-erbα* genes again displayed circadian oscillation in the L18-CAF-GSPE group compared with rats receiving VH. In addition to their functions in the negative feedback loop of the circadian system, these genes have been described to play important roles in lipid metabolism. For instance, *Cry1* inactivation has been shown to reduce adipogenesis in mouse adipocytes [53], whereas *Cry1*-deficient mice show a relative resistance to obesity induced by a high-fat diet, results that were not observed in STD diet-fed animals, highlighting the critical role of the *Cry1* gene under obesogenic conditions [54]. Similarly, *Rev-erbα* is involved in lipid metabolism. In fact, several studies have shown that its deficiency leads to increased hepatic levels of triglycerides and free fatty acids in mice, in addition to increased serum triglycerides and increased BW [55,56,57]. This demonstrates the importance of peripheral tissue clock genes in the improvement of lipid and glucose metabolism [58,59,60,61]. In this regard, serum triglyceride levels were higher in CAF-fed animals under L18 conditions, which do not present significant circadian rhythmicity in any of the genes analyzed, compared to the STD diet-fed group and the CAF-fed group under L6 conditions, which present circadian rhythmicity for the *Cry1* gene in iWAT [22]. Interestingly, the L18-CAF-GSPE group showed reduced serum triglyceride and cholesterol levels [22], which is aligned with the association between GSPE administration and *Cry1* and *Rev-erbα* rhythmicity in iWAT. Regarding L6-CAF-GSPE explants, the *Nampt* gene showed rhythmicity compared to the L6-CAF-VH explants. In fact, this gene has been shown to play a crucial role in insulin sensitivity in mice knockout for this gene in WAT [52]. Interestingly, together with the *Nampt* gene, the *Bmal1* gene tended to show oscillation with GSPE supplementation. This gene not only acts as the main regulator of the molecular clock system, but also plays a key role in glucose metabolism [62,63]. In line with this result, CAF-fed animals under L6 conditions supplemented with GSPE showed significantly lower insulin levels compared to the group that received VH, with both groups showing similar glucose levels [22]. It is well established that GSPE does not exert the same effects in different photoperiods and dietary conditions [23]. Furthermore, our group recently described that the bioavailability of GSPE was photoperiod-dependent, which would explain the variation in its effects [32]. In previous studies, GSPE demonstrated more pronounced effects under L18 conditions at the level of serum biochemical parameters, BW gain, and serum metabolomic profile [21,22]. Interestingly, the iWAT peripheral clock seems to recover its oscillation with GSPE administration under the L18 photoperiod, suggesting a possible reestablishment of peripheral clocks as a prospective therapeutic target for metabolic disorders related to the current lifestyle. Notably, the iWAT explants analyzed in this study were obtained after only one week of photoperiod change to capture the immediate effects of the photoperiod change as an acute disruption, thus minimizing possible adaptation to the new light/dark cycle.

## 5. Conclusions

Based on the results obtained, it has been shown how changing a light/dark cycle to a short photoperiod can alter the circadian oscillation of the iWAT clock genes in both STD- and CAF-fed rats. Although the switch to long photoperiods did not cause an overall loss of rhythmicity in STD-fed rats, the CAF diet induced the genes to dysregulate and thus lose their rhythmicity compared to the STD-fed group. GSPE administration could improve the circadian rhythmicity of the *Nampt* and *Bmal1* genes under a short photoperiod, genes related to insulin sensitivity (Figure 6). Under long photoperiod conditions, GSPE was able to reverse this alteration and show an improvement in the rhythmicity of genes such as *Cry1* and *Rev-erbα*, both related to the regulation of lipid metabolism (Figure 6). Moreover, these results are in concordance with previous results observed in this study, aligning with the improvement of insulin sensitivity and lipid metabolism with GSPE administration in CAF-fed rats under short or long photoperiods. However, further studies are needed to better understand the effects of GSPE under these photoperiods and dietary conditions on circadian oscillation, and also to understand how genes related to energy metabolism are affected by this alteration of clock genes and how this may affect adipose tissue functionality.

## Data Availability

The original contributions presented in this study are included in the article/Supplementary Material. Further inquiries can be directed to the corresponding author.

## Supplementary Materials

The following supporting information can be downloaded at: https://www.mdpi.com/article/10.3390/nu17223564/s1, Figure S1: Experimental design; Table S1: Nucleotide sequences of primers used for PCR amplification in white adipose tissue; Table S2: Circadian oscillations and circadian parameters estimated for *Bmal1* gene expression in inguinal white adipose tissue by Cosinor method; Table S3: Circadian oscillations and circadian parameters estimated for *Cry1* gene expression in inguinal white adipose tissue by Cosinor method; Table S4: Circadian oscillations and circadian parameters estimated for *Per1* gene expression in inguinal white adipose tissue by Cosinor method; Table S5: Circadian oscillations and circadian parameters estimated for *Rev-erbα* gene expression in inguinal white adipose tissue by Cosinor method; Table S6: Circadian oscillations and circadian parameters estimated for *Nampt* gene expression in inguinal white adipose tissue by Cosinor method.
