# Supplementary material for: Grape Seed Flavanols Restore Peripheral Clock of White Adipose Tissue in Obese Rats Under Circadian Alterations
Source: Nutrients. 2025 Nov 14;17(22):3564. doi: 10.3390/nu17223564 (PMC12655575; doi:10.3390/nu17223564)
Supplement: Supplementary file 1 [file nutrients-17-03564-s001.zip › nutrients-3937638-supplementary.pdf]

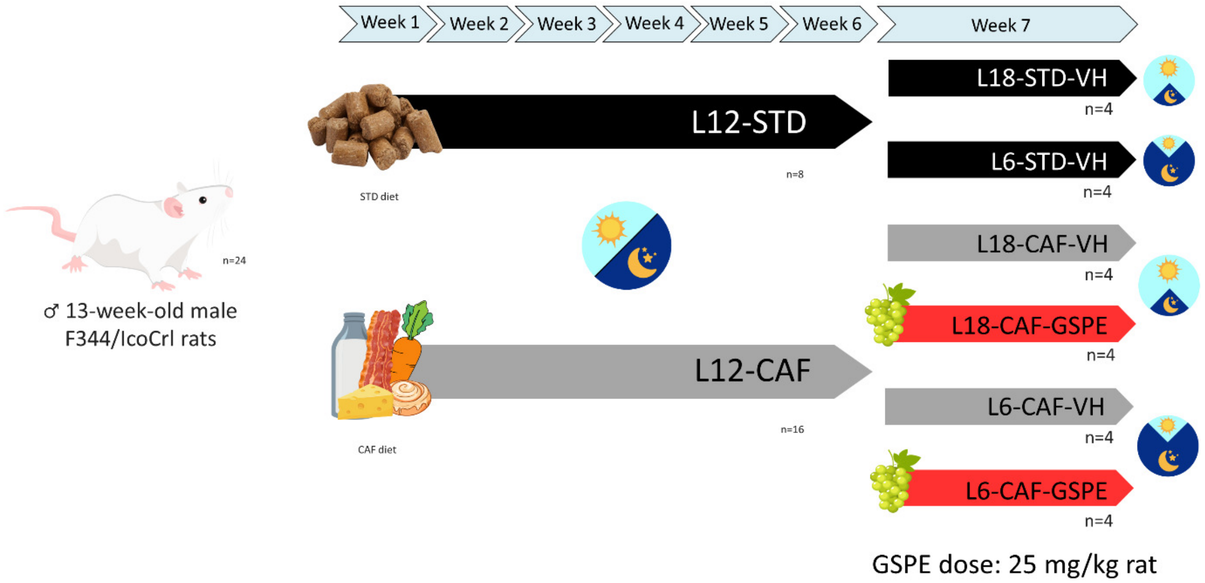

Figure S1. Experimental design. STD- and CAF-fed rats were switched to a new light–dark cycle and VH or GSPE were administered to the animals. STD, L12, photoperiod 12h light per day; L18, photoperiod 18h light per day; L6, photoperiod 6h light per day; CAF, cafeteria diet; STD, standard diet; VH, administered vehicle; GSPE, 25 mg/kg of grape seed proanthocyanidins extract administered.

Table S1. Nucleotide sequences of primers used for PCR amplification in white adipose tissue.

Gene	Forward Primer (5’ to 3’)	Reverse Primer (5’ to 3’)
Bmal1	GGCGTCGGGACAAAATGAAC	AACTTCCGGGACATCGCATT
Per1	CAGTGAGGAGTCTGCTGAGC	GGAGCCTGAAAGTGCATCCT
Cry1	GGACAAGAATCCCGAGGCTC	CATGATGGCGTCAATCCACG
Rev-erbα	GCGTCTGGGTGCTTCATTTC	CGGGTGCAAAAGTCCCAAAG
Nampt	CTCTTCACAAGAGACTGCCG	TTCATGGTCTTTCCCCCACG
Ppia	TCAAACACAAATGGTTCCCAGT	ATTCCTGGACCCAAAACGCT

Bmal1, ARNT-like 1 gene, *Per1*, period family gene, *Cry1*, cryptochrome family gene, *Rev-erbα,* nuclear receptor subfamily 1 group D member 1 gene, *Nampt*, nicotinamide phosphoribosyltransferase gene, *Ppia*, Peptidylprolyl isomerase A gene.

Table S2. Circadian oscillations and circadian parameters estimated for *Bmal1* gene expression in inguinal white adipose tissue by Cosinor method.

Bmal1	Groups						
	Period (h)	Amplitude	CI Amplitude	*p* (amplitude)	Acrophase (h)	CI Acrophase (h)	*p* (acrophase)
L6-STD	24	0.093	-0.040 - 0.228	0.170	3.595	1,688 - 5,501	0.007*
L18-STD	24	0.102	-0.013 - 0.219	0.083#	6.083	20,492 - 15,673	0.577
L6-CAF-VH	24	0.076	-0.040 - 0.193	0.201	2.516	18,913 - 10,118	0.655
L18-CAF-VH	24	0.085	-0.038 - 0.209	0.176	1.552	15,497 - 11,606	0.304
L6-CAF-GSPE	24	0.111	-0.004 - 0.227	0.060#	4.471	0,121 - 8,821	0.001*
L18-CAF-GSPE	24	0.101	-0.026 - 0.228	0.118	2.658	1,444 - 3,872	0.001*

L18, photoperiod 18h light per day; L6, photoperiod 6h light per day; CAF, cafeteria diet; STD, standard diet; VH, administered vehicle; GSPE, 25 mg/kg of grape seed (poly)phenols extract administered; h, hours; Amplitude, difference between the maxim point of the oscillation and the mean value; Acrophase, time (h) when the oscillation reaches its highest point; CI, Confidence Interval. * Indicates significantly parameters ($p < 0.05$). # indicates tendency ($p = 0.1\text{-}0.051$).

Table S3. Circadian oscillations and circadian parameters estimated for *Cry1* gene expression in inguinal white adipose tissue by Cosinor method.

Cry1	Groups						
	Period (h)	Amplitude	CI Amplitude	*p* (amplitude)	Acrophase (h)	CI Acrophase (h)	*p* (acrophase)
L6-STD	24	0.083	-0.091 - 0.258	0.351	22.215	12.814 - 7.607	0.708
L18-STD	24	0.282	0.115 - 0.449	0.001*	0.363	21.696 - 3.031	0.789
L6-CAF-VH	24	0.244	0.064 - 0.425	0.007*	2.766	23.887 - 5.645	0.059#
L18-CAF-VH	24	0.092	-0.103 - 0.288	0.356	6.745	23.799 - 13.690	0.057#
L6-CAF-GSPE	24	0.109	-0.062 - 0.282	0.213	13.647	6.940 - 20.354	0.002*
L18-CAF-GSPE	24	0.123	-0.084 - 0.331	0.088#	4.972	23.643 - 10.301	0.067#

L18, photoperiod 18h light per day; L6, photoperiod 6h light per day; CAF, cafeteria diet; STD, standard diet; VH, administered vehicle; GSPE, 25 mg/kg of grape seed (poly)phenols extract administered; h, hours; Amplitude, difference between the maxim point of the oscillation and the mean value; Acrophase, time (h) when the oscillation reaches its highest point; CI, Confidence Interval. * Indicates significant parameters ($p < 0.05$). # indicates tendency ($p = 0.1\text{-}0.051$).

Table S4. Circadian oscillations and circadian parameters estimated for *Per1* gene expression in inguinal white adipose tissue by Cosinor method.

Per1	Groups						
	Period (h)	Amplitude	CI Amplitude	*p* (amplitude)	Acrophase (h)	CI Acrophase (h)	*p* (acrophase)
L6-STD	24	0.106	-0.118 - 0.330	0.353	17.466	10.603 - 0.331	0.019*
L18-STD	24	0.310	0.119 - 0.501	0.001*	0.689	-2.067 - 3.445	0.026*
L6-CAF-VH	24	0.202	-0.022 - 0.426	0.177	6.393	2.663 - 10.123	0.309
L18-CAF-VH	24	0.199	-0.025 - 0.424	0.082#	6.016	2.376 - 9.657	0.568
L6-CAF-GSPE	24	0.053	-0.138 - 0.246	0.583	14.035	5.833 - 22.237	0.009*
L18-CAF-GSPE	24	0.097	-0.093 - 0.288	0.316	23.630	14.816 - 8.444	0.308

L18, photoperiod 18h light per day; L6, photoperiod 6h light per day; CAF, cafeteria diet; STD, standard diet; VH, administered vehicle; GSPE, 25 mg/kg of grape seed (poly)phenols extract administered; h, hours; Amplitude, difference between the maxim point of the oscillation and the mean value; Acrophase, time (h) when the oscillation reaches its highest point; CI, Confidence Interval. * Indicates significantly parameters ($p < 0.05$). # indicates tendency (p = 0.1-0.051).

Table S5. Circadian oscillations and circadian parameters estimated for *Rev-erbα* gene expression in inguinal white adipose tissue by Cosinor method.

Rev-erbα	Groups						
	Period (h)	Amplitude	CI Amplitude	*p* (amplitude)	Acrophase (h)	CI Acrophase (h)	*p* (acrophase)
L6-STD	24	0.265	0.116 - 0.414	0.001*	14.119	11.809 - 16.429	0.001*
L18-STD	24	0.148	0.006 - 0.291	0.039*	0.627	20.354 - 4.901	0.773
L6-CAF-VH	24	0.030	-0.117 - 0.178	0.681	22.139	18.169 - 2.108	0.855
L18-CAF-VH	24	0.141	-0.004 - 0.287	0.057#	22.377	17.968 - 2.785	0.470
L6-CAF-GSPE	24	0.067	-0.121 - 0.257	0.481	16.519	7.197 - 1.963	0.120
L18-CAF-GSPE	24	0.166	0.009 - 0.323	0.037*	20.704	17.197 - 0.212	0.065#

L18, photoperiod 18h light per day; L6, photoperiod 6h light per day; CAF, cafeteria diet; STD, standard diet; VH, administered vehicle; GSPE, 25 mg/kg of grape seed (poly)phenols extract administered; h, hours; Amplitude, difference between the maxim point of the oscillation and the mean value; Acrophase, time (h) when the oscillation reaches its highest point; CI, Confidence Interval. * Indicates significantly parameters (p < 0.05). # indicates tendency (p = 0.1-0.051).

Table S6. Circadian oscillations and circadian parameters estimated for *Nampt* gene expression in inguinal white adipose tissue by Cosinor method.

Nampt	Groups						
	Period (h)	Amplitude	CI Amplitude	*p* (amplitude)	Acrophase (h)	CI Acrophase (h)	*p* (acrophase)
L6-STD	24	0.156	0.020 - 0.291	0.024*	3.595	0,571 - 6,618	0.019*
L18-STD	24	0.083	-0.05 - 0.216	0.225	6.083	0,700 - 11,465	0.026*
L6-CAF-VH	24	0.099	-0.021 - 0.221	0.107	2.516	21,667 - 7,365	0.309
L18-CAF-VH	24	0.094	-0.023 - 0.210	0.115	1.552	20,219 - 6,884	0.568
L6-CAF-GSPE	24	0.153	0.002 - 0.304	0.047*	4.471	1,115 - 7,828	0.009*
L18-CAF-GSPE	24	0.094	-0.028 - 0.216	0.132	2.658	21,537 - 7,779	0.308

L18, photoperiod 18h light per day; L6, photoperiod 6h light per day; CAF, cafeteria diet; STD, standard diet; VH, administered vehicle; GSPE, 25 mg/kg of grape seed (poly)phenols extract administered; h, hours; Amplitude, difference between the maxim point of the oscillation and the mean value; Acrophase, time (h) when the oscillation reaches its highest point; CI, Confidence Interval. * Indicates significantly parameters ($p < 0.05$). # indicates tendency (p = 0.1-0.051).
